# Bone Regeneration Using Rat-Derived Dedifferentiated Fat Cells Combined with Activated Platelet-Rich Plasma

**DOI:** 10.3390/ma13225097

**Published:** 2020-11-12

**Authors:** Kosuke Nakano, Hirohito Kubo, Masahiro Nakajima, Yoshitomo Honda, Yoshiya Hashimoto

**Affiliations:** 1 Graduate School of Dentistry Department of Oral and Maxillofacial Surgery, Osaka Dental University, 8-1, Kuzuha-hanazono-cho, Hirakata City, Osaka 573-1121, Japan; kosuke-n@cc.osaka-dent.ac.jp; 2 Second Department of Oral and Maxillofacial Surgery, Osaka Dental University, 1-5-17, Otemae, Chuo-ku, Osaka City, Osaka 540-0008, Japan; kubo-h@cc.osaka-dent.ac.jp (H.K.); masa-na@cc.osaka-dent.ac.jp (M.N.); 3 Institute of Dental Research, Osaka Dental University, 8-1, Kuzuha-hanazono-cho, Hirakata City, Osaka 573-1121, Japan; honda-y@cc.osaka-dent.ac.jp; 4 Department of Biomaterials, Osaka Dental University, 8-1, Kuzuha-hanazono-cho, Hirakata City, Osaka 573-1121, Japan

**Keywords:** dedifferentiated fat cells (DFATs), activated platelet-rich plasma (aPRP), bone regeneration

## Abstract

Bone regeneration using mesenchymal stem cells has several limitations. We investigated adipose-derived dedifferentiated fat (DFAT) cells as an alternative, and evaluated their cell proliferation rate, osteoblast differentiation, and bone regeneration ability in combination with activated platelet-rich plasma (aPRP). Rat DFATs and aPRP were isolated using ceiling culture and centrifugation, respectively. The cell proliferation rate was measured, and the cells were cultured in an osteoblast differentiation medium under varying concentrations of aPRP for 21 days and stained with Alizarin red. Gene expression was evaluated using real time polymerase chain reaction. Critical defects were implanted with DFAT seeded gelatin sponges under aPRP, and four weeks later, the bone regeneration ability was evaluated using micro-computed tomography and hematoxylin-eosin staining. The cell proliferation rate was significantly increased by the addition of aPRP. Alizarin red staining was positive 21 days after the start of induction, with significantly higher Runt-related transcription factor 2 (*Runx2)* and osteocalcin (*OCN)* expression levels than those in the controls. A 9 mm critical defect was largely closed (60.6%) after four weeks of gelatin sponge implantation with DFAT and aPRP. Therefore, materials combining DFAT cells and aPRP may be an effective approach for bone regeneration. Further research is needed to explore the long-term effects of these materials.

## 1. Introduction

Bone defects on the maxillofacial surface, caused by congenital abnormalities such as cleft palate, inflammation, tumors, or trauma, cause significant aesthetic and functional challenges [1,2]. To address these issues, research is being directed towards regenerating and treating lost tissues and organs using their constituents [3]. As bone mesenchymal stem cells (BMSCs) are present in the bone marrow and possess the ability to differentiate into mesodermal tissues such as bone, cartilage, and fat, they are clinically used as donor cells for bone regeneration [4,5]. For alveolar reconstruction during dental implant treatment, Ueda et al. [6,7] have developed a bone regeneration method for transplanting mesenchymal stem cells (MSCs) collected from the bone marrow of patients and platelet-rich plasma prepared from autologous blood.

To extend the applicability of regenerative medicine, large quantities of cells that can be prepared directly and with minimal invasion are needed. Therefore, we focused on adipose tissue as a donor cell to replace BMSCs. Excess fat is present in the human body regardless of age or sex and can be collected with minimal invasion. Adipose-derived stem cells (ADSCs) resemble BMSCs and have been obtained. However, BMSCs and ADSCs consist of differing cell populations of vascular endothelial cells, smooth muscle cells, and perivascular cells. In contrast to ADSCs, mature adipocytes are the purest group of cells in the adipose tissue and are obtained by a special method called ceiling culture. These adipocyte-derived fibroblast-like cells are called dedifferentiated adipocytes (DFATs) and have a high proliferation rate and redifferentiation ability [8]. Small adipocytes are reported to be more advantageous for inducing dedifferentiation into DFAT cells than large adipocytes [9,10]. Moreover, DFATs, as with ADSC, are less invasive to collect than BMSCs. 

Platelet-rich plasma (PRP) is a substance that yields platelet-derived growth factor (PDGF) and transforming growth factor β (TGF-β). It is obtained by centrifuging the blood drawn from a patient and combines with BMSCs and gingival connective tissue to promote their growth rate [11,12]. Our previous study [3] demonstrated the potential of the materials, including three-dimensional (3D) woven scaffolds made from biodegradable resins, human BMSC, and PRP, for bone formation in rats. The combined use of PRP and mesenchymal stem cells is considered beneficial for bone regeneration. However, no studies have reported using DFAT in combination with PRP. The purpose of this study is to investigate whether DFATs can replace BMSCs as donor cells to achieve more efficient bone regeneration in conjunction with growth factor-rich PRP.

## 2. Materials and Methods

### 2.1. Animals and Tissue Sampling

The animal experiments in this study strictly conformed to the guidelines approved by the local ethics committee of Osaka Dental University (approval no. 20-04001, approved on 8 June 2020). Seven week old male F344 rats, weighing 250–280 g, were purchased from Shimizu Laboratory Supplies Co. (Kyoto, Japan), and were given solid feed and tap water freely for a week to adapt to the breeding environment. Subsequently, the rats were anesthetized using intraperitoneal injections of a mixture of midazolam (2 mg/kg; Midazolam Sandoz, Sandoz K.K., Yamagata, Japan), medetomidine hydrochloride (0.15 mg/kg; Domitor; Zenoaq, Fukushima, Japan), and butorphanol tartrate (2.5 mg/kg; Vetorphale, Meiji Sika Parma Co., Ltd., Tokyo, Japan). The skin in the groin was incised, approximately 1 g of white fat was obtained, and the wound was closed with a silk thread. Nearly 4 weeks later, blood was collected from the heart using the same anesthesia technique. 

### 2.2. Isolation and Culture of Rat DFATs

**Figure 1:**
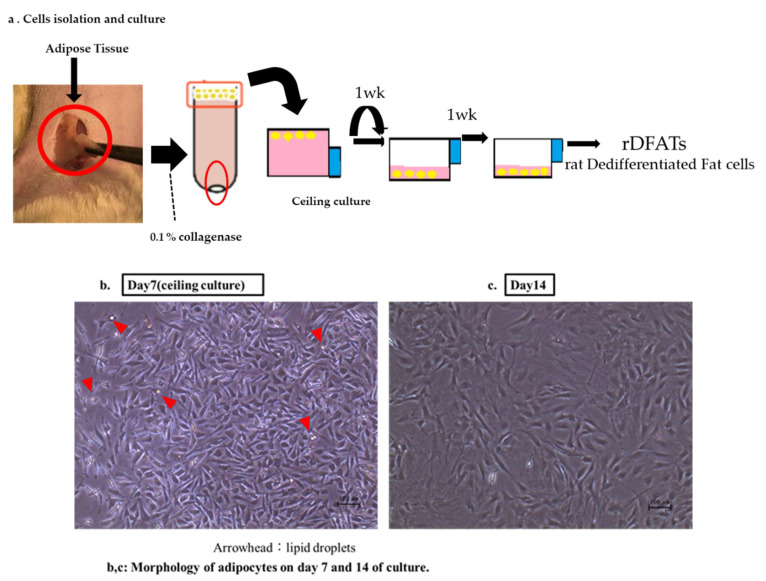
Isolation, dedifferentiation, and culture of mature adipocytes. (**a**) A small piece of adipose tissue was digested with 0.1% collagenase. After centrifugation, unilocular adipocytes were isolated from the floating layer. The isolated cells were cultured in culture flasks filled completely with Dulbecco’s modified Eagle’s medium containing 20% fetal bovine serum for 7 days. During the culture, the cells attached to the upper surface of the flasks and were flattened and converted to fibroblast-like dedifferentiated fat cells (DFATs). Further, the flasks were inverted, and the DFATs were cultured through conventional methods. (**b**) Morphology of adipocytes on day 7 of the culture. The DFATs derived from a unilocular adipocyte (arrowhead) proliferated and formed a colony (dedifferentiation). (**c**) The DFATs rapidly expanded and reached confluence on day 14 of the culture (proliferation).

We isolated rat DFATs (rDFATs) using the ceiling culture method as described in our previous report [13,14]. Briefly, finely minced adipose tissue was digested and dissociated using 0.1% collagenase type I (FUJIFILM Wako Pure Chemical Co. Osaka Japan) for 1 h with gentle stirring. The cell suspension was filtered through 150 µm and 250 µm nylon meshes that allowed the adipocytes to pass through while eliminating the unwanted stromal cells and tissues. The mature adipocytes floating in the top layer were collected and centrifuged at 1000 rpm for 3 min at 4 °C. The isolated mature adipocytes were seeded in a 25 cm² culture flask (Thermo Fisher Scientific Inc., Waltham, MA, USA) filled with Dulbecco’s modified Eagle’s medium (DMEM; Nakalai Tesque, Kyoto, Japan) supplemented with 20% fetal bovine serum (FBS; lot number AUS35775 HyClone). An antibiotic-antifungal mixed stock solution consisting of 10,000 U/mL penicillin, 10,000 µg/mL streptomycin, and 25 µ/mL amphotericin B (Nakalai Tesque) was added to it. The cells were incubated under 5% CO_2_ at 37 °C. The flask was allowed to stand with the adhesive culture surface facing up so that the floating adipocytes containing lipid droplets adhered to the inner surface of the ceiling of the flask. This is the ceiling culture method. A small volume of fresh medium (sufficient to barely cover the bottom of the flask) was added. The medium was then changed twice a week. At confluence, the cells were passaged and used for experiments (Figure 1a–c). We isolated rADSCs according to the method described by Zuk et al. [15].

We isolated activated PRP (aPRP) through a method extensively described in our previous report [16]. In brief, immediately after an intra-abdominal overdose, the rat’s chest was opened and a syringe with an 18 G injection needle (Telmo Co., Ltd., Tokyo, Japan) was used to collect 9.0 mL of blood through cardiac puncture. Further, 7.0 mL of the collected blood was injected into a tube containing 1.0 mL acid-citrate-dextrose solution, formula A, and was tumble mixed. The citrate-treated blood was centrifuged at 1700 rpm for 8 min. Subsequently, the plasma with buffy coat (containing the platelets, leukocytes, and a few erythrocytes) was extracted into a long cannula. The second centrifugation was performed at 3100 rpm for 5 min. Platelet pellets accumulated in the lower part, and the plasma with less platelets accumulated on top. The plasma supernatant was PRP. Platelet pellets in 1.0 mL plasma were used as PRP. The platelets were counted before and after the preparation of PRP. Whole blood—without an anticoagulant—was centrifuged for 8 min at 3500 rpm to collect autologous thrombin. A 1:1 (v/v) mixture of thrombin and calcium chloride was pre-prepared as an activator. A 10:1 (*v/v*) mixture of PRP and the activator was incubated at room temperature for 5 min. This mixture was designated as aPRP. The aPRP was centrifuged at 1000 and 10,000 rpm for 10 min and stored at −80 °C until use. On comparing their concentration ratios, the white blood cell (WBC) count (10^3^/μL), red blood cell (RBC) count (10^6^/μL), and platelet count (10^4^/μL) in whole blood were found to be 5.725, 456.0, and 24.38, respectively; whereas in aPRP, these values were 31.95, 546.8, and 199.2, respectively (Table 1).

### 2.3. Preparation of Gelatin Sponge 

Gelatin (100 mg) from porcine skin (G2500-100G, lot no. SLBB0914V SIGMA, St. Louis, MO, USA) was dissolved in 10 mL Milli-Q water at 70 °C. This solution was poured into a 24-well plate (500 μL/well), and was stored at −30 °C for 24 h. The contents of the plate were lyophilized for 3 days using a DC800 lyophilizer (Yamato Co., Ltd., Tokyo, Japan), and then vacuum-heated using an ETTAS AVO-250NS vacuum dryer (AS ONE, Osaka, Japan). A gelatin sponge (GS) was maintained at a gauge pressure of −0.1 MPa at 150 °C for 24 h [17]. The prepared GS was subjected to dry heat sterilization (180 °C, 2 h) before use. The surface texture of the prepared GS was confirmed. Water was dropped on the surface of the GS and its contact angle with the GS was measured. Furthermore, thermogravimetric and differential thermal analysis (TG/DTA) were performed using a Themo plus EVO (Rigaku, Tokyo, Japan). A heating rate of 5 °C/min was applied in the temperature range of 30–800 °C in air. In the case of TG/DTA, the sample was crushed with a mortar and pestle while freezing in liquid nitrogen, and 4.2 mg of the sample was examined. 

### 2.4. Experiment

#### 2.4.1. Flow Cytometry

Flow cytometry was performed as described in our previous report [18]. At passage 2, rDFATs were suspended in a phosphate buffer solution (PBS; lot no; L6T0348 Nakalai Tesque, Kyoto, Japan) with 0.1% FBS (lot no. AUS35775 HyClone). The cells were immunostained with allophycocyanin and phycoerythrin for 30 min at 4 °C in the dark. The following anti-rat primary antibodies were used: anti-cluster of differentiation (CD)44 PE, anti-CD45 PE, anti-CD90 APC, and anti-CD105 APC. After being washed in PBS with 0.1% FBS, the cells were centrifuged at 1000 rpm for 1 min. The cells were analyzed using fluorescence activated cell sorting (FACS; FACSVerse; BD Biosciences, San Jose, CA, USA). Data were acquired, displayed, and created by Flowjo (BD Biosciences) and the number of positive cells was counted.

#### 2.4.2. Cell Proliferation Assay

After culturing the cells in DMEM for 24 h, the medium was replaced with the experimental medium. The experimental conditions were applied to both the control and experimental groups (10% FBS cells were seeded on a 96-well plate (BD Falcon, Franklin Lakes, NJ, USA) at 2.5 × 10^4^/cell with aPRP weight [wt%]: 3% aPRP, 5% aPRP, 7% aPRP, 10% aPRP). rDFAT in aPRP-added medium (wt%: 3%, 5%, 7%, 10%) was added to Dulbecco’s improved Eagle’s medium. The cells were cultured for 5 days under these conditions, and the proliferation of the cultured cells (cell number) was evaluated using the Cell Counting Kit-8 (Dojindo Molecular Technologies, Gaitersburg, MD, USA), as per the manufacturer’s instructions. The color-developing substrate (WST-8) contained in the Cell Counting Kit-8 is reduced to the water-soluble formazan, which is photometric at 450 nm, by intracellular dehydrogenase, and the amount of formazan in the dish can be directly measured.

#### 2.4.3. In Vitro Differentiation Assay

For osteogenic differentiation, rDFAT was seeded on 96-well plates at 1 × 10^4^ cells/well and cultured to confluence. Cells were cultured for 3 weeks in the osteoblast differentiation-inducing medium (ODM) consisting of DMEM, 10% FBS, 100 nM dexamethasone (Sigma-Aldrich, St. Louis, MO, USA), 10 mM β-glycerophosphate (Sigma-Aldrich, St. Louis, MO, USA), 0.05 mM L-ascorbic acid-2-phosphate (Sigma-Aldrich, St. Louis, MO, USA), and 100 μg/mL bone morphogenetic protein-2 (PeproTech, Rocky Hill, NJ, USA; [19]). The culture media of the experimental group were as follows: (1) DMEM (10% FBS), (2) ODM + DMEM, (3) ODM + DMEM + 3% aPRP (wt%), (4) ODM + DMEM + 5% aPRP (wt%), (5) ODM + DMEM + 7% a PRP (wt%), (6) ODM + DMEM + 10% aPRP (wt%). The medium was changed every 2 days.

#### 2.4.4. RNA Analysis

After 3, 7, and 21 days, the total RNA was isolated using QIAcube PREMIUM (Qiagen, Hilden, Germany) and cDNA was synthesized. The real time polymerase chain reaction (RT-PCR) was conducted using a SuperScriotTM IV VILO Master Mix (Thermo Fisher Scientific) on a Step One Plus PCR system (Applied Biosystems, Foster City, CA, USA) to confirm the expression of Runt-related transcription factor 2 (*Runx2)* (Rn01512298_m1 PN4453320), osteocalcin (*OCN*, Rn0056386_g1 PN4448892), and osteopontin (*OPN*, Rn00681031_m1 PN44448892) using TaqMan®. The PCR conditions were as follows: 2 min at 50 °C and 20 s at 95 °C, followed by 45 cycles of 5 s at 95 °C and 20 s at 60 °C [14]. The obtained data were normalized to GAPDH (Mm99999915_g1) expression levels. The differences in representation between groups were compared using Student’s *t*-test.

#### 2.4.5. Alizarin Red Staining

The plate cultured in ODM for 21 days was collected and the cells were washed with Dulbecco’s PBS (-) after removing the medium and fixed with 95% ethanol (50 μL/well) for 10 min. After removing the fixative, MilliQ water (50 μL/well) was added and removed. Then, 1% Alizarin Red S was added at 50 μL/well and left to stand for 30 min. The stain was removed, and MilliQ water was added at 50 μL/well for washing. This process was repeated six times following which the plate was dried at room temperature.

#### 2.4.6. Cell Seeding into the GS and Scanning Electron Microscopy Analysis

**Figure 2:**
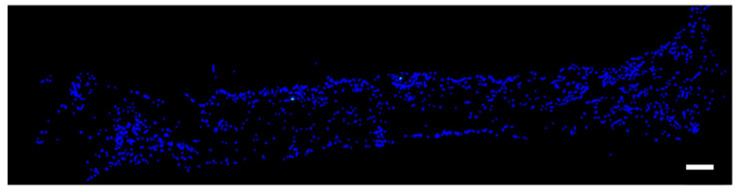
Cells grown on sponges in vitro.The images of rat dedifferentiated adipocytes stained with 4’,6-diamidino-2-phenylindole (DAPI). Bars: 500 μm.

**Figure 3:**
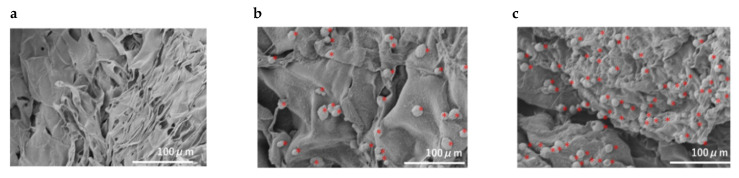
Scanning electron microscopy image. Low and high magnification of SEM images in which the cells were seeded in a gelatin sponge (GS) and cultured for 3 days. The rat dedifferentiated adipocytes (rDFATs) attached to the sponges. (**a**) Cell-free GS, (**b**) GS seeded with rDFATs, (**c**) GS with activated platelet-rich plasma added to rDFATs. Red asterisks show cells.

The prepared rDFATs were cultured in a normal culture, and approximately 2.0 × 10^6^/cells were recovered. Thereafter, a 100 μL suspension was prepared and seeded at 2.0 × 10^6^/cell in the GS placed on a 24-well plate. The plate was centrifuged at 1000 rpm for 3 min. It was left under negative pressure with continuous suction for 30 min. Thereafter, the cells were incubated at 37 °C in 5% CO_2_ for 3 days. Cryosections were prepared from the GS, and an all-in-one fluorescence microscope (BZ-9000) was used to confirm the infiltration of the cells into the GS (Figure 2). Furthermore, the DFAT seeded on the GS was photographed with a scanning electron microscope (SEM; Hitachi High-Technologies field emission scanning electron microscope; S-4800, Tokyo, Japan) (Figure 3). 

#### 2.4.7. Implantation of GS

**Figure 4:**
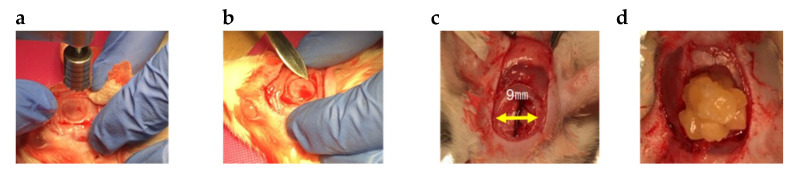
Procedure for sponge implantation (**a**) creation of bone defects using a trephine bar, (**b**) calvaria removal, (**c**) representative bone defects, (**d**) GS implantation.

A critical size defect (9 mm in diameter) was created in the center of the rat calvaria under each condition using a trephine bar (Dentech, Tokyo, Japan). Sterile saline was added intraoperatively to reduce bone damage. The defects were filled with the GS. In the control (no implant and implant GS) group, only saline was added to the defect. The rats were divided into the following groups: 1 = no implant, 2 = GS, 3 = GS + rDFAT, and 4 = GS + rDFAT + aPRP. A total of 20 rats were used in the experiment (5 rats × 4 groups, including a control group without implants). Four weeks after transplantation, the rats in each group were euthanized. The calvaria of each group was collected. After collection, each sample was fixed with a 4% phosphate buffered paraformaldehyde solution (Fujifilm Wako Pure Chemical Industries, Ltd., Osaka, Japan) and evaluated [11] (Figure 4).

#### 2.4.8. Bone Morphometric Analysis

The rats were euthanized after four weeks of transplantation and the calvaria was dissected. Bone regeneration was examined by micro-computed tomography scanning (μCT; SMX-225CT; Kyoto Municipal Institute of Industrial Technology and Culture, Kyoto, Japan). Samples were scanned using a 55 kV tube voltage. The image was saved at a resolution of 512 × 512 pixels. The axial and coronal bone mineral density (BMD) images of the samples were reconstructed using a three-dimensional (3D) image analysis system (TRI/3D-Bon; Ratoc System Engineering, Tokyo, Japan, [20]). Bone formation was measured as bone volume/total volume (BV/TV). BMD was quantified using a cylindrical phantom containing hydroxyapatite (200–1550 mg/cm^3^).

#### 2.4.9. Statistical Analysis

Statistical significance was calculated using Student’s *t*-test. For statistical analysis, Microsoft Excel software was used. All statistical analyses were performed using at least two independent replicas. All results showed high reproducibility.

## 3. Results

### 3.1. Evaluation of the Physical Properties of the GS

**Figure 5:**
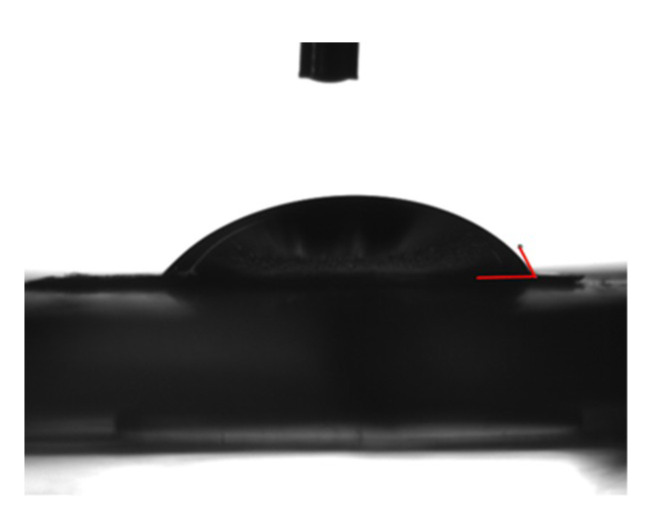
Water contact angle of the gelatin sponge (GS). Macroscopic image. Additionally, 1 μL of water was added dropwise to the GS.

**Figure 6:**
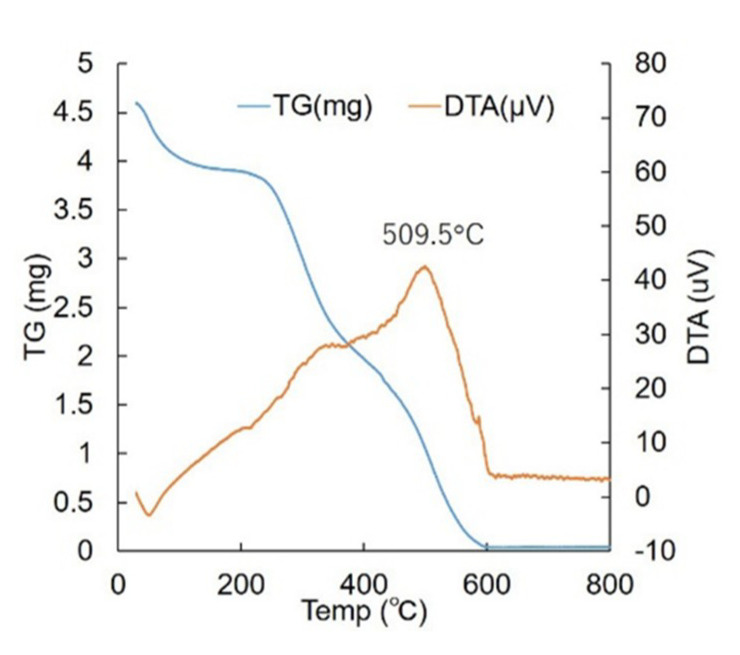
Thermogravimetric analysis and differential thermal analysis (TG/DTA) of the gelatin sponge. The blue line indicates the TGA results and the orange line indicates the DTA results.

When water was dropped on the GS and its wettability was measured, the contact angle was approximately 63° (Figure 5). Thermal analysis of the GS was performed using TG/DTA. Three consecutive drastic weight losses of the GS corresponding to the evaporation of water (30–100 °C), followed by the decomposition (240–360 °C) and combustion (450–550 °C) of the organic matrix were observed in the TGA runs. The high-temperature peak was at 509.5 °C (Figure 6).

### 3.2. Expression of rDFAT Surface Antibodies

**Figure 7:**
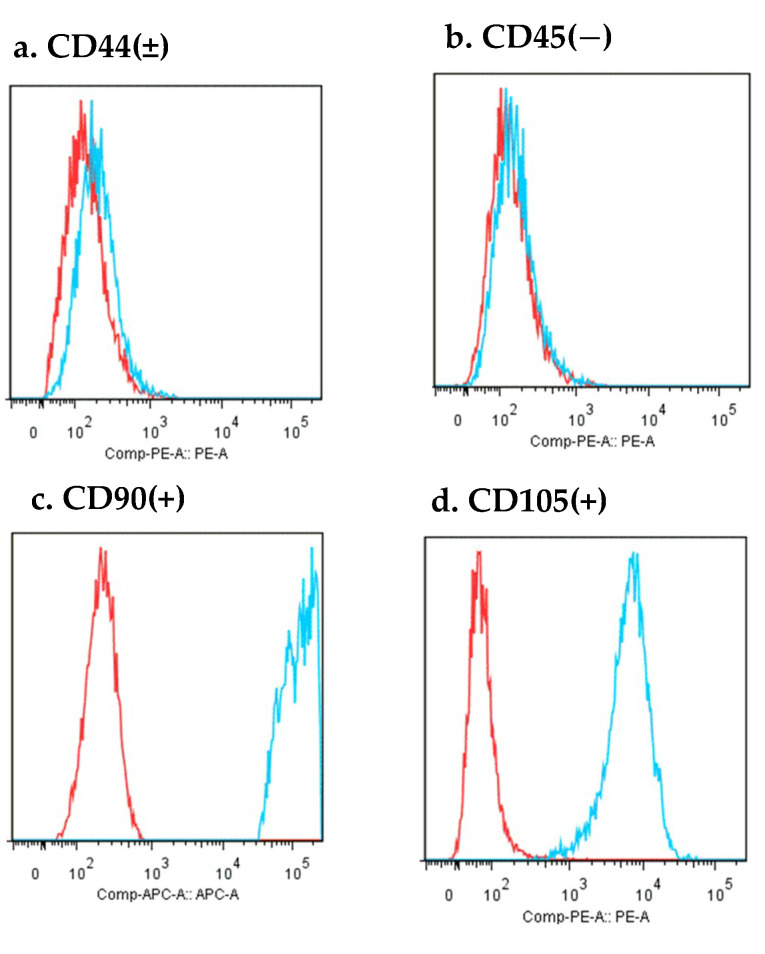
Flow cytometry results for the rat dedifferentiated fat cells, expressing cluster of differentiation (**a**) (CD)44, (**c**) CD90 and (**d**) CD105, but not (**b**) CD45. The red histogram represents the isotype control. The blue histogram represents the positive expression of the CD markers.

The results of the flow cytometry performed on the prepared rDFATs were CD90 (+), CD105 (+), CD44 (±), and CD45 (−) (Figure 7). The CD markers of mesenchymal stem cells were almost the same as those of CD44, 90, 105 being positive and CD45 being negative.

### 3.3. Measurement of Cell Proliferation Rate

**Figure 8:**
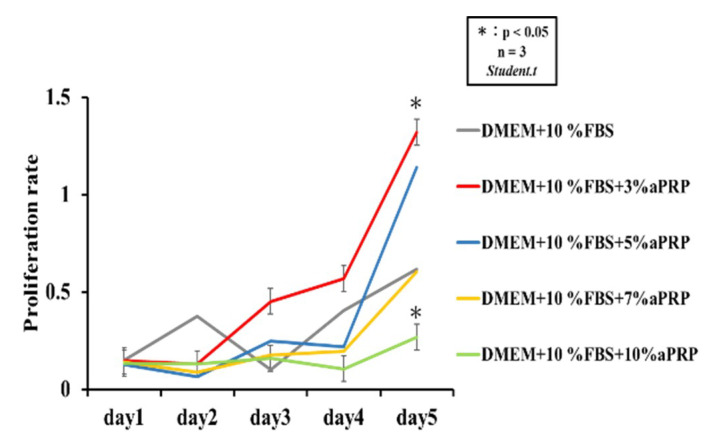
Proliferation of rat dedifferentiated fat cells across 5 days after the addition of various concentrations of activated platelet-rich plasma (aPRP). DMEM: Dulbecco’s modified Eagle’s medium, FBS: fetal bovine serum.

When aPRP was added to the prepared rDFAT and the WST-8 assay was performed, a significant difference was observed between the group supplemented with 3% aPRP and that supplemented with 10% aPRP. The growth rate was the highest in the former group, and the latter resulted in a lower proliferation rate than the DMEM (10% FBS) (Figure 8).

### 3.4. Alizarin Red staining

**Figure 9:**
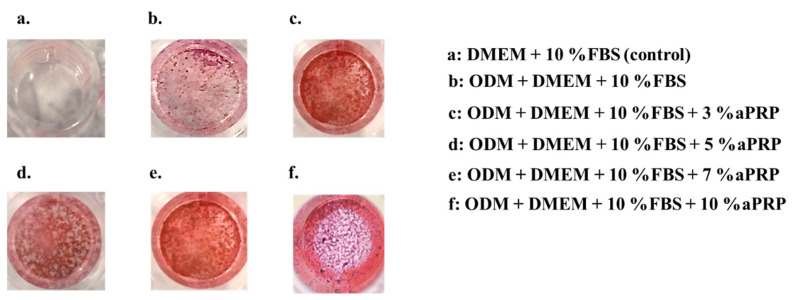
Twenty-one days after Alizarin red staining: (**a**) Dulbecco’s modified Eagle’s medium (DMEM) + 10% fetal bovine serum (FBS) (control), (**b**) osteoblast differentiation-inducing medium (ODM) + DMEM + 10% FBS, (**c**) ODM + DMEM + 10% FBS + 3% activated platelet-rich plasma (aPRP), (**d**) ODM + DMEM + 10% FBS + 5% aPRP, (**e**) ODM + DMEM + 10% FBS + 7% aPRP, and (**f**) ODM + DMEM + 10% FBS + 10% aPRP.

Staining of the calcified granules was observed in all experimental groups, but not in the control group (Figure 9).

### 3.5. Expression of Osteoblast Differentiation Markers

**Figure 10:**
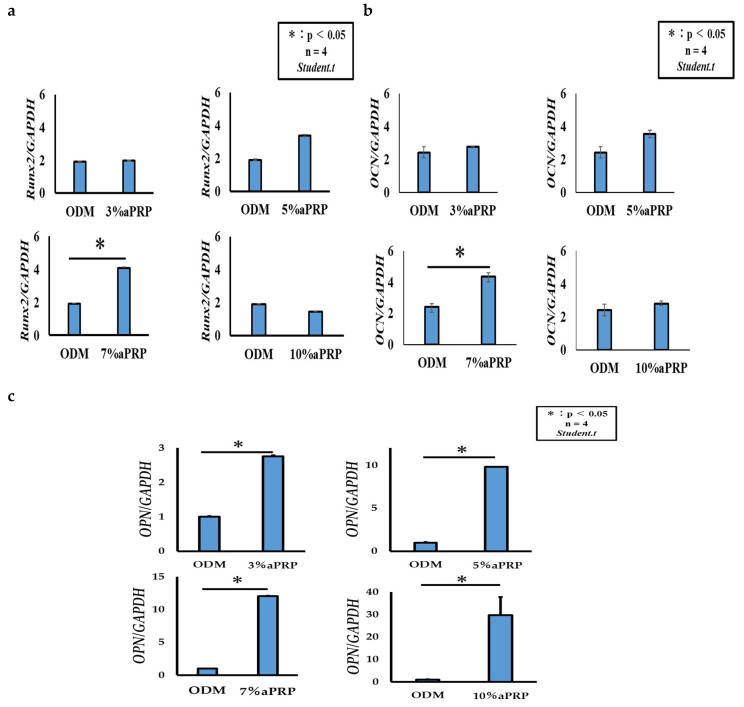
Gene expression of Runt-related transcription factor 2 (*Runx2)*, osteocalcin (*OCN),* and osteopontin (*OPN)* during dedifferentiation of rat dedifferentiated fat cells. (**a**) The expression level of *Runx2:* osteoblast induction day 3. (**b**,**c**) The expression level of *OCN*, *OPN:* osteoblast induction day 21.

RT-PCR showed that the expression level of *Runx2*—an early marker of bone differentiation—was elevated in rDFATs under the conditions of ODM + 7% aPRP-supplementation on the third day. These expression levels decreased under 10% aPRP-supplementation; however, no significant difference was observed between the 3% aPRP-supplementation conditions and those compared exclusively with ODM (Figure 10a). Further, a similar increase in the expression of *OCN*—a late marker of bone differentiation—was observed under the condition of 7% aPRP-supplementation. A significant difference was observed in *OCN* expression levels under exclusive ODM conditions, unlike *Runx2* (Figure 10b). In addition, *OPN*, which is a late marker of bone differentiation similar to *OCN*, showed particularly higher expression levels under all the conditions tested with aPRP addition compared to those with ODM alone (Figure 10c).

### 3.6. Bone Formation in a Rat Calvarial Defect Model

**Figure 11:**
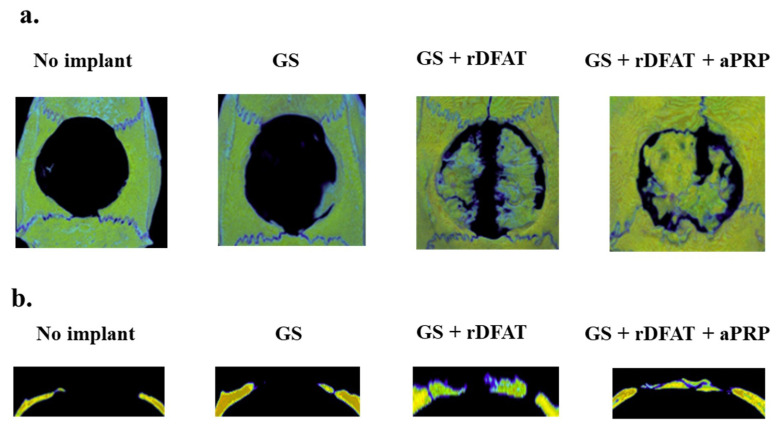
Micro-computed tomography analysis and microscopic images of the defects. Representative macroscopic images of the defects treated with sponges after implantation surgery at 4 weeks. (**a**,**b**) Axial and coronal bone mineral density (BMD) image, reconstructed by a three-dimensional image analysis system, of the defects treated with or without the sponges after 4 weeks of implantation. GS: gelatin sponge, rDFAT: rat dedifferentiated fat cells, aPRP: activated platelet-rich plasma.

A piece of sponge was implanted in the calvaria defect to evaluate the bone-forming ability of each condition (Figure 11). Shown is a microcomputer tomography image (the axial and coronal planes) reconstructed by a three-dimensional (3D) image analysis system four weeks after implantation. Bone formation was shown in conditions treated with GS + rDFAT + aPRP rather than conditions treated with GS + rDFAT or GS and no implant. It was revealed that the radiation permeability of the implant was greater than that of the defect (Figure 11a,b). 

**Figure 12:**
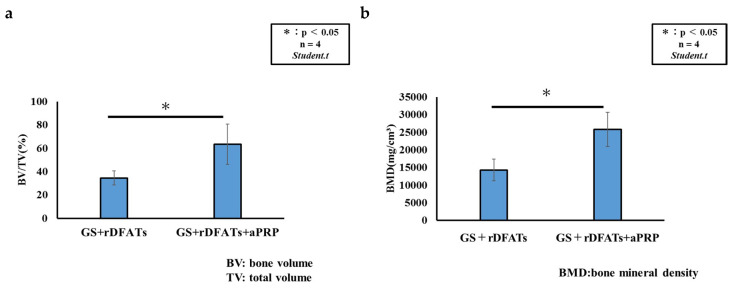
Morphometric analysis of bone defects. Quantitative analysis of micro-computed tomography data for each defect. GS: gelatin sponge, rDFATs: rat dedifferentiated fat cells, aPRP: activated platelet-rich plasma (**a**) BV: bone volume; TV: total volume. (**b**) BMD: bone mineral density.

Bone defects transplanted with GS + rDFAT and GS + rDFAT + aPRP had significant differences in BMD, and the BV and bone mineral contents per total volume for the latter were significantly greater than those for the former (Figure 12a,b). It is noteworthy that the samples using GS + rDFATs + aPRP almost completely closed 9 mm (critical size) defects within 4 weeks without the addition of other materials. A 9 mm critical defect was largely closed (60.6%) after four weeks of GS implantation with DFAT and aPRP. Bone quality and bone morphology were measured using the Ratoc system. A difference was observed between the GS + rDFAT and the GS + rDFAT + aPRP at BV/TV and BMD.

### 3.7. Pathological Evaluation

**Figure 13:**
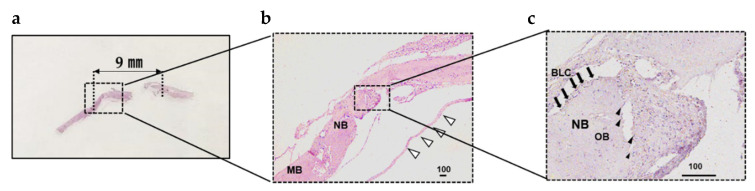
Histological images of the defect treated with sponges. (**a**) Created bone defect (9 mm). (**b**) Low-magnification image. (**c**) High magnification histological image. MB: mother bone. NB: newly formed bone. OB: osteoblast (arrowheads). BLC: bone lining cell (arrows). White arrows: integrated fibrous matrix of GS. Bars: 100 μm for lowest magnification images of (**b**,**c**).

Figure 13 shows the histology of the defect treated with GS + rDFAT + aPRP 4 weeks after transplantation. Fiber structure was detected in defects treated with GS + rDFATs + aPRP (arrows in the high magnification diagram in Figure 13c), with many cells attached to the fibrous matrix. These matrices were incorporated into the newly formed bone (Figure 13b). At the margin of the defects, osteoconduction occurred from the native bone (Figure 13b,c). In addition, both activated osteoblast-like cells (cubic shape) and multinucleated cells (osteoclast-like cells) were found in the newly-formed bone of the defect treated with GS + rDFATs + aPRP. Bone lining cells, which are thinly extended over new bone surfaces, were observed (Figure 13c), showing that bone remodeling persisted after 4 weeks.

## 4. Discussion

DFAT cells have recently been shown to differentiate into multiple mesodermal lineages, including osteoblasts [8]. DFATs were uniformly positive for CD44, CD90, CD105, but negative for CD45 (Figure 7). This profile is consistent with a previous finding for bone marrow MSCs [21]. The cell-surface antigen profile of ADSCs at passage one was essentially the same as that of DFAT cells. It has been reported that ADSCs are a heterogeneous population, which includes smooth muscle cells, endothelial cells, mast cells, and lineage-committed progenitor cells [21]. They resemble MSCs and ASDCs in terms of cell markers and proliferative capacity, but are more homogenous [8,22], and can differentiate into osteoblasts much earlier [13,23]. However, the evidence regarding the use of DFAT in combination with PRP to induce bone tissue engineering is inadequate. In this study, we provide evidence to fill this knowledge gap by evaluating cell proliferation, osteoinduction, and bone regeneration using rDFAT and aPRP in vitro and in vivo. 

aPRP is a substance enriched with platelets and various growth factors and is suggested to facilitate healing by controlling the local inflammatory response [24,25]. Moreover, its addition is expected to induce more efficient cell proliferation and early bone differentiation. A possible explanation for this could be that the PDGF and TGF-β released from aPRP cause hypertrophy and enhanced cell proliferation. According to the results of this experiment, the optimum aPRP concentration for cell proliferation was considered to be 3%, and when the aPRP concentration was higher than 10%, cell proliferation was suppressed. Graziani et al. reported that the high aPRP effect was achieved in 2.5 times of the concentration, with higher concentrations resulting in a reduction in cell proliferation [26]. Similarly, Choi et al. [27] examined the influence of PRP concentrations on the viability and proliferation of alveolar bone cells and showed that these factors were suppressed at high PRP concentrations (10% or more), but stimulated by low PRP concentrations (1–5%); this supports our results. However, a concentration of 3% aPRP is too low for bone differentiation, and it is considered that an aPRP with a high concentration is required to induce bone differentiation. Therefore, it was considered that the induction of differentiation into osteoblasts was promoted at an aPRP concentration of 7% or more. Over 7% aPRP supplementation was associated with increased levels of *Runx2* expression. In addition, *OCN* and *OPN* showed significant differences under these concentration conditions 21 days after the start of bone induction in rDFAT cells. This time lag and impact on gene expression levels occurred in rDFAT cells that comprise a cell population of high-purity mature adipocytes, but not in rADSCs that contain other cells such as vascular endothelial cells, smooth muscle cells and perivascular cells [21]. Therefore, we infer that that the sensitivity to aPRP varies, suggesting that its concentration for efficient cell proliferation and induction of bone differentiation may differ according to the cell types it is used in conjunction with. 

Our results demonstrate that GS + rDFATs + aPRP showed better bone-forming ability than GS + rDFATs, indicating that the effect of aPRP was retained when aPRP was used on rDFAT cells. Notably, the rats in our experimental GS + rDFATs + aPRP group closed a large-sized defect (9 mm) in the calvaria in 4 weeks, as seen in the axial view of the BMD. In a previous study using a similar bone defect model, the BV/TV value of the bone defect after 4 weeks was about 37.5% when autologous bone was transplanted, compared to 60.6% when GS + rDFATs + aPRP was transplanted.

Bone renewal requires angiogenesis to supply cells and nutrients, and bone is regenerated by the cells and nutrient supply from blood vessels. Since bone regeneration in rat parietal bone is caused by membranous ossification, the angiogenesis during bone regeneration is theorized to begin from existing blood vessels in the surrounding tissues. Matsui et al. [28] and Den et al. [29] reported that after implantation of octacalcium phosphate or interconnected porous calcium phosphate into the subperiosteal pocket of rat calvaria, new bone formation was recognized from the calvarial portion and not from the periosteal portion, as opposed to the findings in the present study. A possible explanation offered was that the elevation of the periosteum reduced its osteogenic potential [29,30]. Furthermore, rDFATs are known to differentiate into osteoblasts. However, the rate of bone growth could depend on the degree of cross-linking of the GS scaffold [31,32]. Considering that bone chondral regeneration occurred when rDFATs were transplanted into the defect [33], the rDFATs may have proliferated and differentiated into endovascular cells and osteoblasts by being stimulated by the tissues surrounding the bone defect [34,35], causing bone formation. Moreover, the vascular endothelial cells may have proliferated due to the action of the PDGF contained in aPRP. Therefore, the effective osteogenesis may be a result of combining the osteoinducing ability of aPRP and the differentiation ability of rDFATs. In addition, during the post-operative local inflammation occurring in the wound area, the release of inflammatory cytokines such as TNF-α may interfere with bone regeneration by stimulating osteoclast activity [36]. However, administration of IFN-γ suppresses TNF-α expression and promotes bone regeneration, suggesting that the anti-inflammatory effect of rDFATs in conjunction with aPRP aids in bone regeneration. These findings suggest that GS + rDFATs + aPRP culminates to offer a promising candidate for bone regeneration.

In conclusion, our study clearly indicates the value of using rDFATs in conjunction with aPRP for bone regeneration. However, further analyses of this material—particularly by tracking its fate in long-term implantations—are imperative to verify its potential application, mechanism of bone formation, safety, and the degree of GS cross-linking. Detailed studies that carefully examine the above-stated concerns may prove beneficial in understanding the clinical application of this material.

## Figures and Tables

**Table 1:** Comparison of blood count and concentration: whole blood and activated platelet-rich plasma (aPRP).

	WBC (10^3^/µL)	RBC (10^6^/µL)	Platelet (10^4^/µL)
Whole Blood	5.725	456.0	24.38
aPRP	31.95	546.8	199.2
Concentration Rate	×5.58	×1.20	×8.17
